# Autoimmune Thyroiditis Presenting as Palmoplantar Keratoderma

**DOI:** 10.1155/2010/604890

**Published:** 2010-03-14

**Authors:** Sara Lestre, Eva Lozano, Cláudia Meireles, Ana Barata Feio

**Affiliations:** ^1^Dermatology Department, Hospital de Santo António dos Capuchos, 1150-314 Lisboa, Portugal; ^2^Internal Medicine Department, Hospital de Santo António dos Capuchos, 1150-314 Lisboa, Portugal

## Abstract

Palmoplantar keratoderma is a heterogeneous group of hereditary and acquired disorders characterized by abnormal thickening of palms and soles. Hypothyroidism is an unusual cause of palmoplantar keratoderma, rarely reported in the literature. We report a case of a 43-year-old woman presented with a 3-month history of a diffuse palmoplantar hyperkeratosis unresponsive to topical keratolytics and corticosteroids. Her past medical and family histories were unremarkable. She complained of recent asthenia, mood changes and constipation. Laboratory evaluation revealed an autoimmune thyroiditis with hypothyroidism. Other causes of acquired palmoplantar keratoderma were excluded. After hormonal replacement therapy institution, a gradual improvement of skin condition was observed. The diagnosis of underlying causes for acquired palmoplantar keratoderma can be a difficult task; however its recognition is essential for successful treatment results. Although a very rare association, hypothyroidism must be suspected in patients with acquired palmoplantar keratoderma, particularly when it occurs in association with systemic symptoms.

## 1. Introduction

Palmoplantar keratoderma is a heterogeneous group of hereditary and acquired disorders characterized by abnormal thickening of palms and soles. Acquired palmoplantar keratoderma has been associated to numerous underlying causes, such as psoriasis, lichen planus, pityriasis rubra pilaris, eczema, Reiter's syndrome, fungal infections, keratoderma climactericum, trauma, drugs, chemicals and malignancies [1–4]. 

Thyroid disorders have a high prevalence in medical practice. Hypothyroidism is a common endocrine disorder resulting from deficiency of thyroid hormones, most frequently caused by autoimmune thyroiditis. Many of the most common symptoms and signs of hypothyroidism are nonspecific [5]. The hypothyroid state is associated with a wide variety of cutaneous findings [6]; however, its association with palmoplantar keratoderma is seldom described in the medical literature.

## 2. Case Presentation

**Figure 1:**
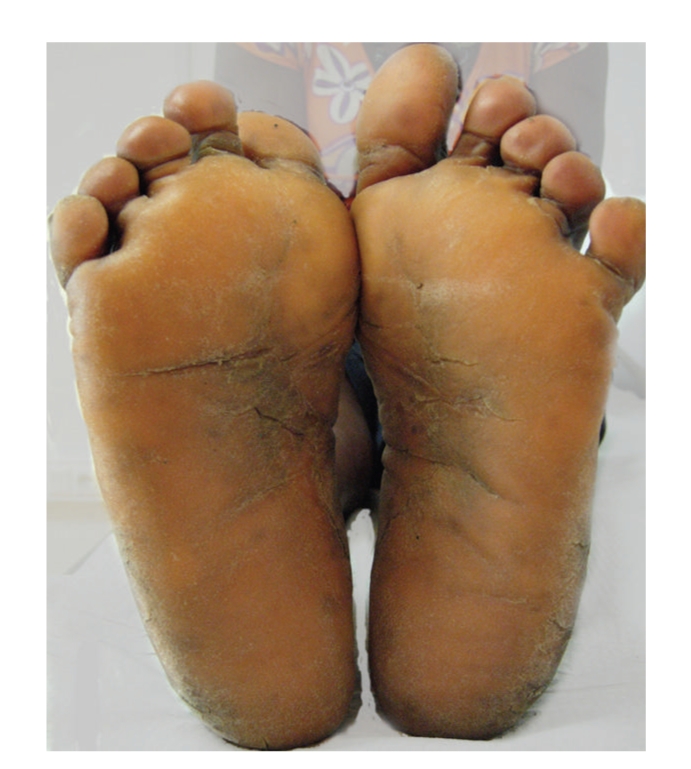
Diffuse plantar keratoderma, with painful fissures.

**Figure 2:**
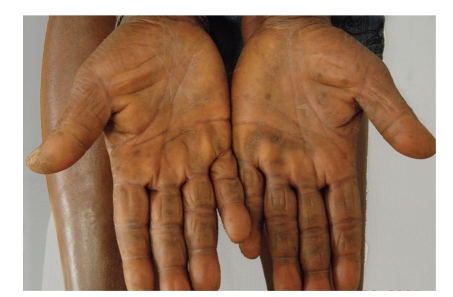
Diffuse palmar keratoderma with a yellowish hue.

A 43-year-old black woman presented with a 3-month history of symmetric, diffuse and yellowish hyperkeratosis of palms and soles. Plantar surfaces were the most affected, with painful fissures (Figures 1 and 2). A progressive worsening was seen in the last 2 months, despite treatment with topical antifungals, oral terbinafine and topical corticosteroids. Dry skin with generalized pruritus was also observed. Neck examination was unremarkable. The remainder physical examination (including nails, mucosae and hair) was normal. Asthenia, mood changes with emotional lability, constipation and menstrual irregularities were present for the last 4 months. Her past medical history was unremarkable. She denied the use of any medication. Family history was negative for dermatological diseases.

A complete laboratory and imaging evaluation was performed in order to investigate possible infectious, systemic, or malignant conditions. Routine laboratory tests were normal, except for elevated total cholesterol (295 mg/dL) and LDL cholesterol (205 mg/dL). Thyroid function tests revealed an elevated thyroid stimulating hormone (17,5 U/mL; normal range 0,35–4,94) and diminished free triiodothyronine (1,45 pg/mL; normal range 1,71–3,71) and free thyroxine (0,46 ng/dL; normal range 0,7–1,48). Circulating antibodies to thyroid peroxidase were elevated (534 U/mL) and thyroglobulin antibody was negative. Other immunological tests (rheumatoid factor, antinuclear antibodies, antidouble stranded DNA antibodies, antiextractable nuclear antigen antibodies and complement) were also normal or negative. Infections by hepatitis B and C viruses, human immunodeficiency virus 1 and 2, *Treponema pallidum* and intestinal parasites were excluded. Fungal cultures from skin and nails were negative. Tumor markers (CEA, CA 19-9, CA-125, CA 15-3, *β*2-microglobulin and AFP) were normal. Abdominal and pelvic ultrasound, mammography and chest radiograph were unremarkable. A thyroid ultrasound showed a homogeneous ultrasonographic thyroid image and excluded nodules and goitre. The patient refused to give consent for a skin biopsy.

**Figure 3:**
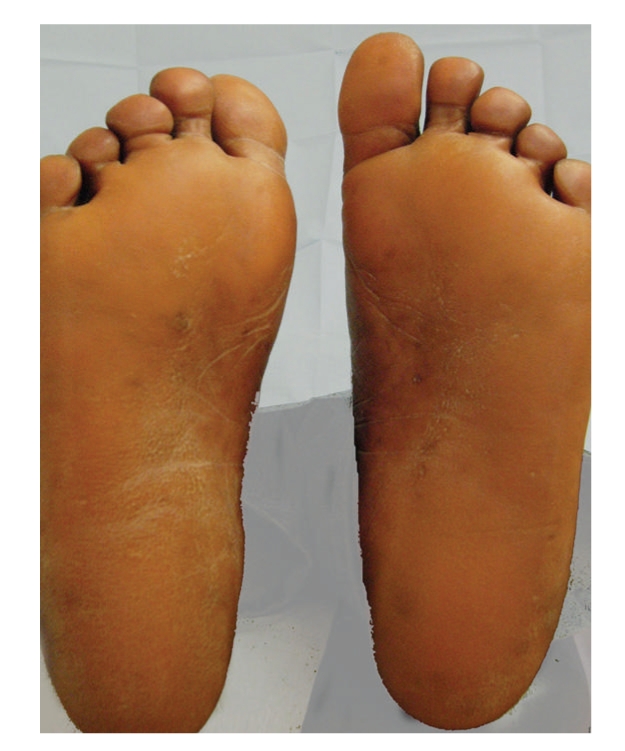
Improvement of plantar keratoderma after 9 months of levothyroxine therapy.

**Figure 4:**
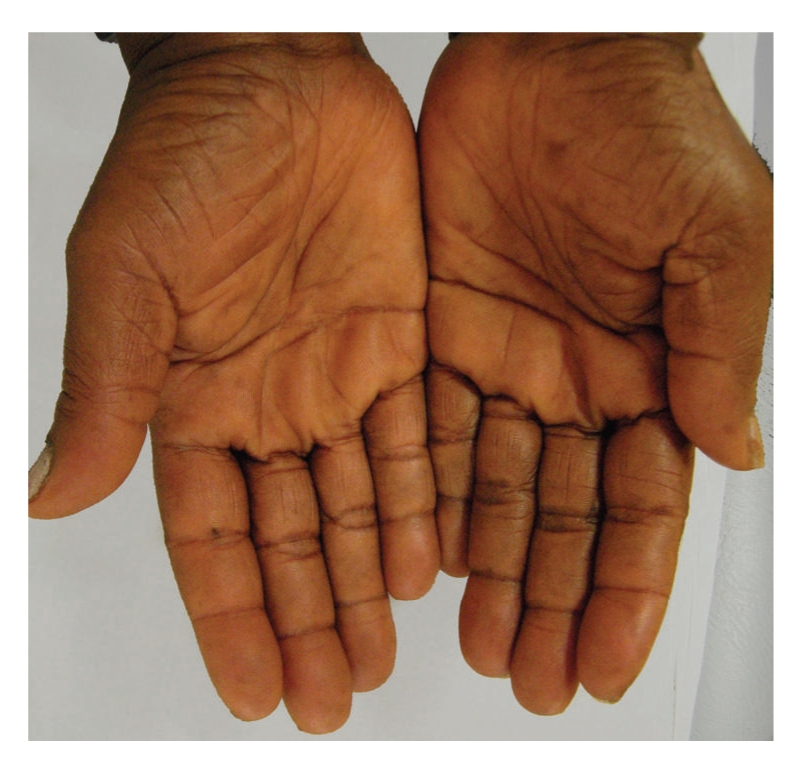
Complete clinical remission of palmar hyperkeratosis at 9 months of levothyroxine therapy.

Initially, empirical treatment with emollients, topical keratolytics (urea, salicylic acid) and isotretinoin 20 mg/day was started. Despite those treatments, worsening of palmoplantar keratoderma was observed. Skin xerosis improved after treatment with topical emollients. Later, with the diagnosis of primary hypothyroidism secondary to autoimmune thyroiditis, the patient was referred to Internal Medicine Department and therapy with levothyroxine (0, 1 mg/day) was started. A slow and progressive improvement was seen, with partial and complete clinical remission of cutaneous findings at 3 and 9 months of hormonal replacement therapy, respectively (Figures 3 and 4). Systemic symptoms had also an excellent and sustained response to the treatment. After 24 months of follow-up, she continues levothyroxine with normal thyroid function tests and no recurrences of the dermatosis.

## 3. Discussion

Palmoplantar keratodermas are a diverse group of disorders characterized by thickening of the skin of the palms and soles caused by excessive keratin. The palmoplantar keratodermas can be divided based on whether they are inherited or acquired. Hereditary palmoplantar keratodermas tend to occur in infancy, with positive family history (autosomal dominant or recessive) and can be accompanied by associated features (lesions of nonvolar skin, hair, teeth, nails, sweat glands and/or abnormalities of other organs) [7, 8]. Acquired palmoplantar keratoderma can be defined as a nonhereditary, non-frictional hyperkeratosis of the palms and/or soles that involves more than half of the surface of involved acral areas [1]. Just as the hereditary forms, it can be classified into 3 clinical patterns of epidermal involvement: diffuse, focal and punctuate. Acquired palmoplantar keratoderma is a multietiological disorder; therefore, a comprehensive history and complete physical examination are essential in the diagnostic approach of these patients.

The functioning of the skin depends on the general status of the body and it is controlled by hormones, like the thyroid. Hypothyroidism can be associated with dry skin, hypohidrosis, generalized myxedema, purpura, ecchymosis, xantomas, carotenodermia, pruritus and pyodermitis [6, 9]. Autoimmune thyroiditis, also called Hashimoto thyroiditis, is a thyroid disease that occurs as a result of an immune response directed against the thyroid gland. It is the most frequent cause of hypothyroidism. Laboratorial findings include increased thyroid stimulating hormone (TSH), low thyroxine, hypercholesterolemia and the presence of thyroid auto-antibodies [10].

Hypothyroidism has been rarely associated with palmoplantar keratoderma. To our knowledge, only 8 cases have been reported in the literature [11–17]. This uncommon association was first described in 1952 by Shaw et al. [11]. In 1977, Tan and Sarkani [12] described myxedema with palmar keratoderma. Later, in 1986, Hodak et al. [13] reported a case of a 63-year-old female with a 13-year history of intractable severe hyperkeratosis of the hands and feet associated with myxedema. A striking improvement was seen 3 months after institution of substitution therapy with thyroid hormone. Since then, 5 similar cases have been reported [14–17]. In the majority of the patients, hypothyroidism was caused by autoimmune thyroiditis [14, 15, 17]. Distinctive clinical features of keratoderma include a yellowish hue, marked severity, diffuse involvement of the soles and more limited palmar involvement, lack of response to topical corticosteroids and keratolytics and a rapid response to thyroid hormone replacement. Histopathological findings have been unspecific, with marked hyperkeratosis and acanthosis. In contrast to all previous published cases, in which myxedema was present, in our case the clinical examination was normal, except for the presence of palmoplantar keratoderma and dry skin. The systemic complaints of our patient, such as asthenia, constipation and menstrual irregularities were unspecific and could be easily attributed to the aging process and/or to psychological stress. In all previous reports, a lack of response to topical corticosteroids and keratolytics was also observed. This fact contrasts with the excellent clinical response to thyroid hormonal replacement, with complete clinical remission after 1 month [14] to 9 months of therapy [15]. Similarly, in our case, a sustained clinical response to levothyroxine treatment was observed, supporting a causal relationship between hypothyroidism and palmoplantar keratoderma. Its etiopathogenesis remains not well understood, but it may be associated with a disturbance of intercellular stratum corneum lipids [15].

Acquired palmoplantar keratoderma due to hypothyroidism is a reversible condition and its recognition is essential for successful treatment results. Although a rare association, hypothyroidism should be considered in the differential diagnosis of acquired palmoplantar keratoderma.
